# Fluorescent DNA Biosensor for Single-Base Mismatch Detection Assisted by Cationic Comb-Type Copolymer

**DOI:** 10.3390/molecules24030575

**Published:** 2019-02-05

**Authors:** Jialun Han, Jincai Wu, Jie Du

**Affiliations:** State Key Laboratory of Marine Resource Utilization in South China Sea, College of materials and chemical engineering, Hainan University, Haikou 570228, China; jialun_han@126.com (J.H.); 13698954926@163.com (J.W.)

**Keywords:** fluorescent DNA biosensor, SNP analysis, single-base mismatch detection, cationic comb-type copolymer, graphene oxide

## Abstract

Simple and rapid detection of DNA single base mismatch or point mutation is of great significance for the diagnosis, treatment, and detection of single nucleotide polymorphism (SNP) in genetic diseases. Homogeneous mutation assays with fast hybridization kinetics and amplified discrimination signals facilitate the automatic detection. Herein we report a quick and cost-effective assay for SNP analysis with a fluorescent single-labeled DNA probe. This convenient strategy is based on the efficient quenching effect and the preferential binding of graphene oxide (GO) to ssDNA over dsDNA. Further, a cationic comb-type copolymer (CCC), poly(l-lysine)-*graft*-dextran (PLL-*g*-Dex), significantly accelerates DNA hybridization and strand-exchange reaction, amplifying the effective distinction of the kinetic barrier between a perfect matched DNA and a mismatched DNA. Moreover, in vitro experiments indicate that RAW 264.7 cells cultured on PLL-*g*-Dex exhibits excellent survival and proliferation ability, which makes this mismatch detection strategy highly sensitive and practical.

## 1. Introduction

The simple and rapid detection of DNA single base mismatch and point mutation is of great significance for the diagnosis, treatment, and identification of single nucleotide polymorphism (SNP) in genetic diseases [1]. Many detection techniques, including allele-specific hybridization, allele-specific primer extension, and oligonucleotide ligation, are being used to identify SNP alleles [2,3]. However, there are still some unsolved practical issues in these methods. For example, they require sufficient and specific hybridization of the primer or probe DNA to the target DNA. This often requires optimization of the primer/probe structure and operating conditions [4]. Therefore, homogeneous mutation assays with fast hybridization kinetics and amplified discrimination signals facilitate the automatic detection.

In a previous study, DNA mutation detection was achieved by monitoring the kinetic differences in the strand exchange reactions (SERs) between double-strand (ds) DNA probes and single-strand (ss) target DNA [5,6,7]. The basic principle is to determine that there is a mismatch between the slower SER rate and the perfectly matched target. In these measurements, cationic copolymers acting as SER accelerators allow for the effective differentiation of kinetic barriers between perfectly matched DNA and mismatched DNA without careful optimization of the process [5]. However, these assays use a dsDNA probe labeled with a pair of fluorescence resonance energy transfer (FRET) fluorophores to detect SER. Since the preparation of dual-labeled probes is both cumbersome and expensive, simple, fast, and low-cost probes are needed to make mismatch detection more practical.

As a water-soluble derivative of graphene, graphene oxide (GO) has great potential in biosensors and biological analysis. [8,9,10,11,12,13,14,15]. Compared with other nanomaterials, GO is low cost, with low fluorescence background owing to the high FRET efficiency between the GO and the fluorophores, and a high signal-to-noise ratio. More especially, it is well known that GO can effectively quench the fluorescence of organic dyes through long-range nanometer energy transfer. In addition, it is reported that GO strongly interacts with ssDNA nucleotides through the π-stacking between the ring structure of the nucleobase and the hexagonal cells of GO, while dsDNA cannot be adsorbed stably on the GO surface because it effectively shields the nucleotides in negatively charged dsDNA phosphoric acid backbone [16]. Therefore, GO can interact with ssDNA noncovalently by π-π stacking interactions between nucleotide bases and GO, but hardly interact with rigid dsDNA or aptamer-target complexes. Moreover, GO can protect DNA against enzymatic cleavage compared with free DNA probes. Based on these properties of GO mentioned above, a series of convenient and universal strategies were explored for highly sensitive, highly selective, and low-cost detection of DNA [8,14,15], metal ions [17], small molecules [9], protein [18], the screening of aptamers [19], and even in situ cellular imaging [20,21]. However, the target DNA-sequence signal specificity is very low, thus the rate of formation and release of dsDNA from GO is very slow, and the single base mismatch target cannot be distinguished, which prevents the limitation of detection from being further improved. Recently, DNA chips and microarrays using water-soluble cationic conjugated polymers were reported to increase the sensitivity of fluorescent DNA biosensors [22,23,24].

In nature, highly homologous hybridization is assisted by nucleic acid chaperones, which reduce energy barriers associated with breaking and recombination of nucleotide pairs [4,5]. Here, we report a simple, rapid, accurate and enzyme-free SNP analysis method. The method uses cationic comb-type copolymer (CCC) to produce high nucleic acid chaperone activity. Inter-polyelectrolyte complexes between cationic polymers and DNA strands are of interest not only as model systems but also as non-viral therapeutic gene-transfer systems. However, electroneutral inter-polyelectrolyte complexes lead to precipitation and aggregation and are, therefore, not suitable for stabilizing the multilevel DNA structures. A synthetic copolymer consisting of a polycationic backbone with hydrophilic graft chains can be used to quantify the structure and stability of DNA, because the hydrophilic chains make the complexes water soluble. In our previous studies, we demonstrated that CCC composed of polycationic backbone and abundant hydrophilic graft (>90 wt%) affected the kinetics and thermodynamics of nucleic acid hybridization under physiological conditions. In addition, we found that the poly-(l-lysine)-*graft*-dextran (PLL-*g*-Dex) significantly accelerates DNA hybridization [25]. Moreover, the copolymer remarkably promoted the self-assembly of DNA blocks so that there was no need for the annealing process, while stabilized the assembled multi-level structures relative to that in buffer alone [26]. The efficiency of DNA building blocks is the foundation of the bottom-up development of three-dimensional DNA hydrogels. Further, we found that in presence of PLL-*g*-Dex, DNA hydrogels were easily obtained under physiological conditions within one minute, and the critical gelation concentration of DNA hydrogels was significantly reduced even with short sticky end, the mechanical properties of DNA hydrogels were also improved obviously [27]. The foundation of our research is that CCC reduces an entropically unfavorable counterion condensation effect that accompanies a nucleation process of SER, lowering the energy barrier associated with breakage and reassociation of nucleic acid base pairs. Detailly, The Dex graft chains on the copolymer might inhibit the close contact of DNAs to the PLL backbones and thereby dehydration and compaction, which are presumably involved in the irreversible complex formation between DNAs and homopolycations. Regardless of the weakened interaction, the comb-type copolymer could suppress the repulsive forces among DNA strands enough to stabilize the multilevel DNA structure. In addition to the shielding effect upon the repulsive forces, Dex chains may play a role in stabilizing hydrogen bonding between base pairs. The DNAs that are attracted to the PLL backbone by electrostatic interactions are forced to merge with the Dex-enriched phases, which are low in dielectric constant. Such low dielectric environments might enhance hydrogen bonding between base pairs, leading to DNA hydrogel stabilization.

In this work, we examined the activity of CCC to assist GO based fluorescent DNA biosensor for single-base mismatch detection. The purpose of this study was to design a simple, quick, and cost-effective assay for SNP analysis with a single-labeled DNA probe. This convenient strategy is based on the preferential combination of GO and ssDNA rather than dsDNA, which combines with the efficient quenching effect. In addition, cationic comb-type copolymer PLL-*g*-Dex significantly accelerates DNA hybridization and SER, allowing an effective distinction between fully matched DNA and DNA containing mismatches, which would endow this mismatch detection strategy as highly sensitive and practical.

## 2. Materials and Methods

### 2.1. Reagents and Materials

**Figure 1:**
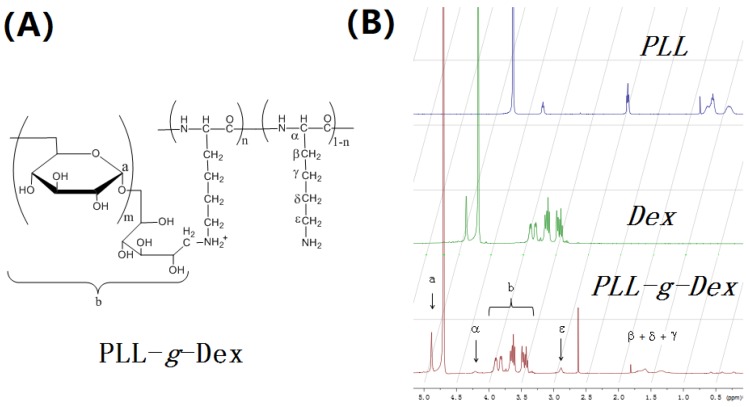
(**A**) Structural formula of poly(l-lysine)(PLL)-*g*-Dex. (**B**) ^1^H-NMR spectra of PLL, Dex and PLL-*g*-Dex in D_2_O. The dextran content of the copolymer was calculated from ^1^H-NMR signals assigned to PLL (ε-CH_2_) and dextran (C_1_-H, a).

All oligonucleotides were supplied by FASMAC Co., Ltd. (Midorigaoka, Atsugi, Kanagawa, Japan) and purified by reverse-phase high performance liquid chromatography. GO was supplied by Key Laboratory of Advanced Materials of Tropical Island Resources, Ministry of Education (Haikou, China). Other chemical reagents were purchased from Sigma-Aldrich (Shanghai, China). The sequence of T-DNA, cDNA, M1, M2, M3 were detailed listed in Table 1. Cationic comb-type copolymer PLL-*g*-Dex (*M*_n_ = 65,000) was prepared by a reductive amination reaction of PLL·HBr (*M*_n_ = 20,000, BACHEM Shinagawa, Tokyo, Japan) with dextran (*M*_n_ = 5,900, Dextran T-10, Amersham Pharmacia Biotech, Beijing, China) as described previously [28]. The dextran content of the copolymer was 91 wt%, determined by ^1^H-NMR (Figure 1). DNA samples were treated by annealing (heating to 95 °C for 5 min, and then quick cooling on ice).

### 2.2. Fluorescence Spectroscopy

DNA solution was dissolved in sodium phosphate buffer (10 mM sodium phosphate, 0.5 mM EDTA, 150 mM sodium chloride, pH 7.2). The concentration of the stock solution of the DNA was 20 nM. Firstly, the baseline emission value was recorded for about 5 min, then the DNA solution was added into the syringe. The change in fluorescence intensity of the mixture in a 10 mm square quartz tube was recorded by a JASCO FP-6500 fluorescence spectrometer (JASCO, Osaka, Japan) with a Peltier thermostatic control cell holder at excitation and emission wavelengths of 560 and 582 nm, respectively.

### 2.3. UV-Melting Point (T_m_) Measurement

The final DNA concentration in the sodium phosphate buffer (10 mM sodium phosphate, 0.5 mm EDTA, 150 mM NaCl, pH 7.2) was 2 μM. The DNA mixture solution was heated at 95 °C for 3 min, and then gradually cooled to room temperature. The UV spectrum at 260 nm was recorded by a Shimadzu UV-1650 PC spectrometer equipped with a TMSPC-8 temperature controller (Shimadzu, Kyoto, Japan). The melting curve was obtained by a heating rate of 0.5 °C/min and a cooling rate of 0.5 °C/min. The inflection point of the melting curve of absorbance intensity at 260 nm vs. the temperature is determined by the melting point of the DNA sample. The differential absorbance (ΔA = A_260_ − A_340_) was calculated to correct for baseline shift. The first derivative [d(ΔA)/dT] was calculated from the melting curve data. Peak temperatures in the derivative curves were designated T_m_ [28].

### 2.4. Cell Culture

Commercial rat macrophages (RAW 264.7 cells) were used for cell culture. PLL-*g*-Dex was placed in a 24-well plate, sterilized with 75% (*v*/*v*) ethanol for 150 min, then rinsed with sterilized phosphate buffer saline (PBS) three times. Subsequently, PLL-*g*-Dex was pre-wetted with a culture medium for 120 min. After removing the culture medium, 500 μL RAW 264.7 cell suspension (1 × 10^5^ cells well^−1^) was inoculated directly as a control on the PLL-*g*-Dex or culture plate and cultured under standard conditions. After incubation for 24 h, PLL-*g*-Dex or cells on the culture plate were stained with Live/Dead assay kit, and then observed with a fluorescence microscope. Cell counting kit-8 (CCK-8) was used to evaluate cell proliferation in PLL-*g*-Dex or culture plate 1, 3, 5, and 7 days after culture. In short, at each time point, the medium was removed and the CCK-8 working solution was added at 37 °C for 120 min. Finally, the supernatant culture medium was extracted by Thermo Scientific Enzyme Marker (Waltham, MA, USA) to detect the number of cells.

## 3. Results and Discussion

**Scheme 1:**
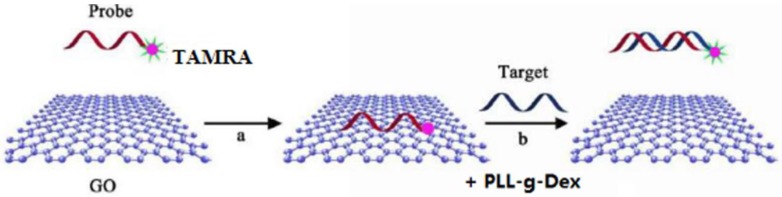
Principle for the graphene oxide (GO) based fluorescent DNA biosensor for single-base mismatch detection assisted by cationic comb-type copolymer.

In order to increase the sensitivity and practicality of the single-base mismatch detection, in this work a DNA biosensor system was constructed by utilizing the difference of binding ability of GO to single-stranded over a double-stranded DNA. The hybridization of the complementary or mismatched strands with the DNA probe is accelerated, and thus the fluorescent changes are amplified, chaperoned by a cationic copolymer PLL-*g*-Dex. Combined with the highly efficient quenching effect of GO, herein a convenient and versatile strategy is explored for rapid, highly sensitive, and low-cost single-base mismatch detection. The principle is shown in Scheme 1.

**Figure 2:**
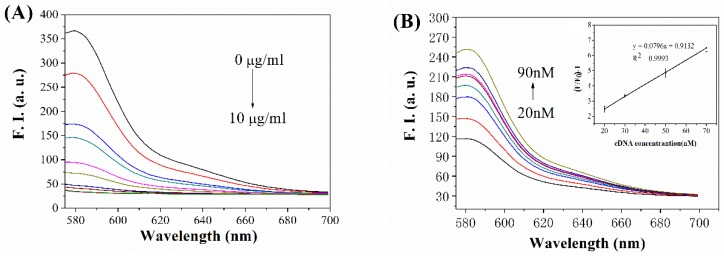
(**A**) The fluorescence intensity of TAMRA labled T-DNA (20 nM) in the presence of various concentrations of GO (0, 2, 3, 4, 5, 6, 7, 8, 9, 10 μg/mL); (**B**) the fluorescence intensity of T-DNA-GO in the presence of different concentrations of cDNA (20, 30, 40, 50, 60, 70, 80, and 90 nM); (insert) the values of [(F/F_0_)-1] for assay with the concentration of cDNA.

Firstly, the quench efficiency of GO binding ssDNA was detected, as shown in Figure 2A. The fluorescence intensity of TAMRA-labeled T-DNA (6 μL, 20 nM) changes with the different concentrations of GO (0, 2, 3, 4, 5, 6, 7, 8, 9, and 10 μg/mL). It was shown that the fluorescent intensity of T-DNA was gradually decreased with the increasing GO concentration. When the concentration of GO is 9 μg/mL, the fluorescent intensity of DNA is quenched approximately 91.6%. While the concentration of GO is below 9 μg/mL, the fluorescent intensity of DNA cannot be quenched enough to achieve the purpose of detection. When the concentration of GO is higher than 9 μg/mL, excessive GO causes more fluorescent DNA to bind on the surface of GO, which would weaken the sensitivity of further detection. Thus, the concentration of GO in the following work is fixed at 9 μg/mL.

A series of cDNAs with different concentrations (20, 30, 40, 50, 60, 70, 80, and 90 nM) were added into the sensor system to detect the fluorescence recovery of the mixture of 20 nM T-DNA and 9 μg/mL GO mentioned above. As shown in Figure 2B, when the concentration of cDNA increased from 20 nM to 90 nM, the fluorescence of T-DNA-GO system continuously recovered. The reason is that the higher the concentration of cDNA contained in the system, the greater the hybridization chance with fluorescent T-DNA. Meanwhile, the hybridization force between cDNA and T-DNA is greater than the binding force of T-DNA on GO, and thus T-DNA is desorbed from the surface of GO, hybridizing with cDNA, and the fluorescence intensity subsequently recovers. When the concentration of cDNA increased to 90 nM, the fluorescent intensity was 251.05, restored up to 69.2%. The high sensitivity is due to the superior quenching ability of GO, as well as the low background and high signal-to-noise ratio.

**Figure 3:**
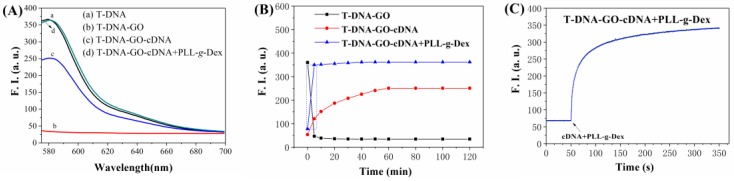
(**A**) The fluorescence intensity of T-DNA (20 nM) under the following conditions: (a) T-DNA in the solution; (b) T-DNA + GO (9 μg/mL); (c) T-DNA + GO (9 μg/mL) + cDNA (90 nM); and (d) T-DNA + GO (9 μg/mL) + cDNA (90 nM) + PLL-*g*-Dex (96 nM). (**B**) The fluorescence intensity of T-DNA (20 nM) with reactive time under the following conditions: (■) T-DNA + GO (9 μg/mL); (●) T-DNA + GO (9 μg/mL) + cDNA (90 nM); (▲) T-DNA + GO (9 μg/mL) + cDNA (90 nM) + PLL-*g*-Dex (96 nM). (**C**) The fluorescence intensity changes in (B) T-DNA + GO (9 μg/mL) + cDNA (90 nM) + PLL-*g*-Dex (96 nM) within the first 300 s hybridization.

Figure 3A describes the fluorescence spectra of T-DNA under different conditions. When 20 nM T-DNA is present in PBS buffer alone, the system emitted a strong fluorescence with value of 363. After adding GO solution, T-DNA binds to the surface of GO through the π-stacking between the ring structure of nucleobase and the hexagonal cells of GO. Thus, the fluorescent intensity of T-DNA is quenched at almost 91.6% by GO. Adding a fully matched cDNA (96 nM) into the system, the double-stranded DNA appears by the hybridization of the two single-strands of DNA during a period of time. Because the double-stranded DNA base pairs in nucleic acid structure do not significantly interact with GO, and thus maintain the double helix structure. Therefore, the fluorescence intensity recovery can be detected. The algorithm of fluorescence intensity recovery rate is that the constant fluorescence intensity value F of each system subtracts the fluorescence intensity value F_0_ of T-DNA-GO, and the difference is then divided by the fluorescence intensity value of T-DNA separately in PBS buffer. Notice that, after adding cDNA into the T-DNA-GO quenched system, the fluorescence intensity slowly recovers up to only 60% after one hour, and cannot reach full recovery, as shown in Figure 3B. While in the presence of 70 nM PLL-*g*-Dex, the fluorescence intensity is restored up to approximately 90.0% within 300 s (Figure 3C). The results illustrate that PLL-*g*-Dex promotes DNA hybridization while stabilizing DNA double strands. 

**Figure 4:**
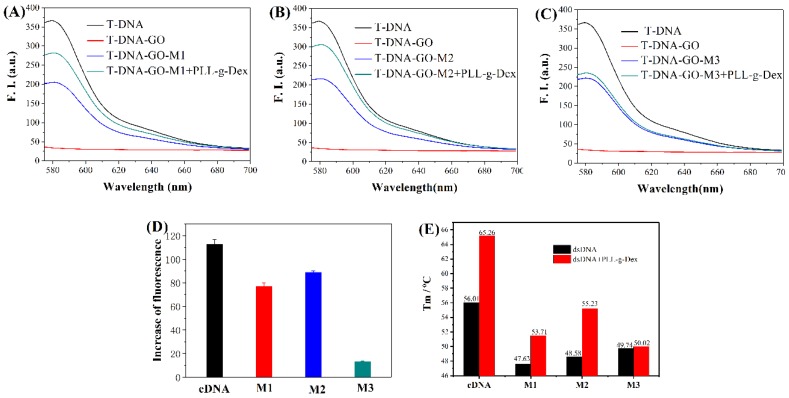
(**A**–**C**) The fluorescence intensity of target chains with different mismatched bases. (**D**) The increase of fluorescent intensity at 582 nm in the presence of PLL-*g*-Dex, compared with the fluorescent intensity at 582 nm without PLL-*g*-Dex. (**E**) T_m_ of different double-strands DNA without (black) or with (red) PLL-*g*-Dex. [GO (9 μg/mL), cDNA, M1, M2, or M3 (90 nM), PLL-*g*-Dex (96 nM)].

Based on above results, a simple, quick, and cost-effective assay for SNP analysis with single-labeled DNA probe could be designed via GO based fluorescent DNA biosensor assisted by cationic comb-type copolymer. The fluorescence spectra of T-DNA-GO (20 nM) were examined after adding mismatched different single-stranded DNA: M1, M2 and M3. When complementary cDNA (96 nM) was added into the T-DNA-GO system, the fluorescence intensity could be restored to approximately 60.0% (Figure 3A), while that could be restored to only 47.2% (Figure 4A), 50.3% (Figure 4B), and 51.6% (Figure 4C) when M1, M2, and M3 were added at the same concentration. Therefore, we could use fluorescent detection technology through the enhancement of fluorescence intensity to identify base mismatch in target DNA. The enhancement of fluorescence intensity was: cDNA > M3 > M2 > M1, that is, C-G > C-C > C-A > C-T, indicating that the DNA biosensor could be used for detection of single-base mismatch. 

In the presence of PLL-*g*-Dex, all of the fluorescence intensity recovered more, due to the chaperone activity of cationic copolymer. Figure 4D summarized the increase value of fluorescence intensity in each cationic comb-type copolymer assisted system, showing that enhancement of fluorescence intensity: cDNA > M2 > M1 > M3, that is, C-G > C-A > C-T > C-C. These data revealed that the recovery of M3 system containing C-C base mismatch is the smallest, while that of other systems containing cDNA, M1 and M2 increased more. The results showed that PLL-*g*-Dex had the best stabilization effect on the perfectly matched cDNA, but the C-A and C-T mismatch could be restored partly because the mismatch position in the DNA sequence was in the middle position. On this basis, in the presence of cationic copolymer, the fluorescent intensity recovered more, also indicating that the stability of the double-strands was increased. For the C-C base mismatch system, the fluorescence did not increase significantly, indicating that these mismatched double-strands were not stable even with the cationic copolymer. Compared with other bases, PLL-*g*-Dex has strong selectivity for the mismatched target. The chaperone activity of PLL-*g*-Dex could also be evaluated by UV-T_m_ analysis. Table 2 summarized the melting temperature of the dsDNA samples by UV-T_m_ measurement, recorded during heating and cooling. The corresponding average T_m_ were also shown as a bar graph in Figure 4E, the results demonstrated that the copolymer increased T_m_ of the dsDNA, and thus stabilizing the multi-level DNA structure. The enhancement of T_m_: cDNA > M2 > M1 > M3, with the similar tendency as that in Figure 4D. Notice that, in this work PLL-*g*-Dex significantly accelerates DNA hybridization at low DNA concentration, allowing an effective distinction between fully matched DNA and DNA containing mismatches, which would endow this mismatch detection strategy highly sensitive and practical. The limit of target detection in this work is 10 nM of fully matched DNA and 20 nM of single mismatched DNA using 96 nM of PLL-*g*-Dex, more sensitive than many other work [14,15].

**Figure 5:**
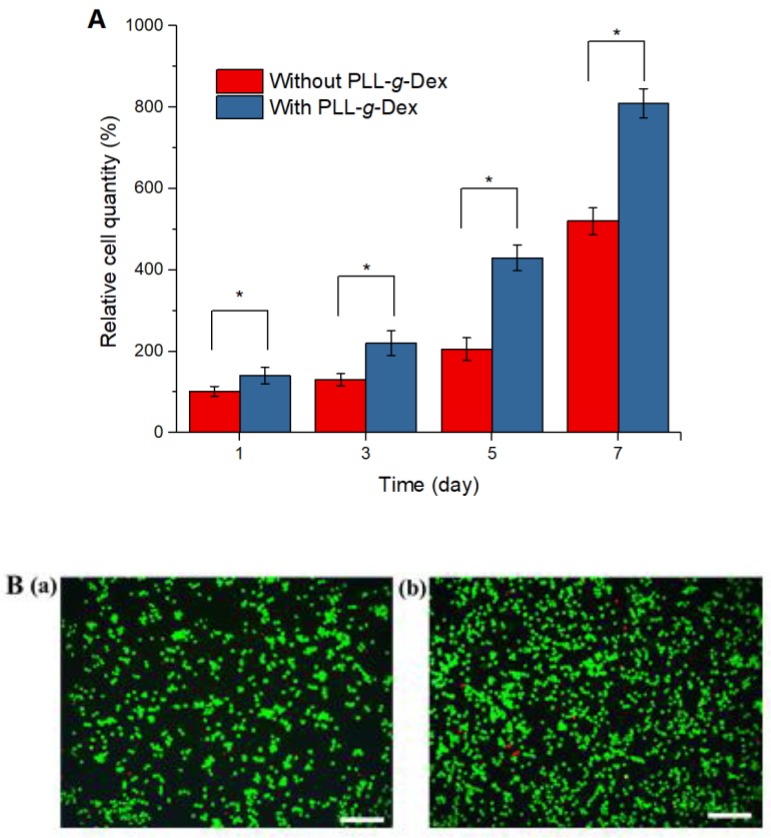
(**A**) Proliferation of the RAW 264.7 cells cultured on the surfaces of the PLL-*g*-Dex or the culture plates (without PLL-*g*-Dex). * *p* < 0.05. (**B**) Viability and morphology of the RAW 264.7 cells cultured on surfaces of (a) the culture plates and (b) PLL-*g*-Dex for 24 h (green cells are live, and red cells are dead; the scale bar is 200 μm).

The effects of PLL-*g*-Dex on the survival and proliferation of RAW 264.7 cells cultured were studied in vitro. With the increasing culture time, RAW 264.7 cells gradually proliferated in the plates with or without PLL-*g*-Dex (Figure 5A). The proliferation rate of RAW 264.7 cell with PLL-*g*-Dex was greater than that without PLL-*g*-Dex. Cell viability was also researched using a Live/Dead Assay Kit. All samples showed high cell viability (green) (Figure 5B), but more cells were observed on PLL-*g*-Dex, indicating that PLL-*g*-Dex showed good viability and proliferation ability. Therefore, PLL-*g*-Dex has good cell compatibility and potential application value in vitro.

## 4. Conclusions

In conclusion, we successfully designed a quick, sensitive, and cost-effective assay for SNP analysis with a single-labeled DNA probe. Cationic comb-type copolymer, PLL-*g*-Dex, significantly accelerated DNA hybridization while stabilizing the double-stranded DNA. The hybridization of the complementary or mismatched strands with the DNA probe is accelerated, and thus the fluorescent changes are amplified, chaperoned by PLL-*g*-Dex. The limit of target detection in this work is 10 nM of fully matched DNA and 20 nM of single mismatched DNA using 96 nM of PLL-*g*-Dex. The increase value of fluorescence intensity in each cationic comb-type copolymer assisted system, showing the enhancement of fluorescence intensity: C-G > C-A > C-T > C-C. From this point of view, different single-base mismatches could be easily detected by this sensitive fluorescent DNA biosensor. Moreover, RAW 264.7 cells cultured on PLL-*g*-Dex showed good viability and proliferation ability. Therefore, PLL-*g*-Dex with good biocompatibility will potentially be a highly sensitive and practical platform for the diagnosis, treatment of genetic diseases, and identification of single nucleotide polymorphisms. More complex cell experiments, especially the SNP detection in live cells, are in progress and will be submitted later. 

## Figures and Tables

**Table 1:** DNA sequence.

Oligonucleotide	Sequence
T-DNA	5′-TAMRA-TCTTTGGGACCACTGTCG-3′
cDNA	5′-CGACAGTGGTCCCAAAGA-3′
M1 (C-T)	5′-CGACAGTGTTCCCAAAGA-3′
M2 (C-A)	5′-CGACAGTGATCCCAAAGA-3′
M3 (C-C)	5′-CGACAGTGCTCCCAAAGA-3′

**Table 2:** T_m_ of different double-strands DNA without or with PLL-*g*-Dex.

Double-Strands DNA	T_m_ (without PLL-*g*-Dex)			T_m_ (with PLL-*g*-Dex)		
	Heating	Cooling	Average	Heating	Cooling	Average
T-DNA/cDNA	54.68	57.34	56.01	64.15	66.37	65.26
T-DNA/M1	46.85	48.41	47.63	52.68	54.74	53.71
T-DNA/M2	47.68	49.48	48.58	54.86	55.60	55.23
T-DNA/M3	49.45	50.01	49.74	49.65	50.39	50.02

## References

[1] Kashuk C., SenGupta S., Eichler E., Chakravarti A. (2002). ViewGene: A graphical tool for polymorphism visualization and characterization. Genome Res..

[2] Brennan M.D. (2001). High throughput genotyping technologies for pharmacogenomics. Am. J. Pharm..

[3] Kwok P.Y. (2001). Methods for genotyping single nucleotide polymorphisms. Annu. Rev. Genom. Hum. Genet..

[4] Chicurel M. (2001). Faster, better, cheaper genotyping. Nature.

[5] Kim W.J., Sato Y., Akaike T., Maruyama A. (2003). Cationic comb-type copolymers for DNA analysis. Nat. Mater..

[6] Kim W.J., Akaike T., Maruyama A. (2002). DNA strand exchange stimulated by spontaneous complex formation with cationic comb-type copolymer. J. Am. Chem. Soc..

[7] Li Q., Luan G., Guo Q., Liang J. (2002). A new class of homogeneous nucleic acid probes based on specific displacement hybridization. Nucleic Acids Res..

[8] Lu C.H., Yang H.H., Zhu C.L., Chen X., Chen G.N. (2009). A graphene platform for sensing biomolecules. Angew. Chem. Int. Ed..

[9] Jang H.J., Kim Y.K., Kwon H.M., Yeo W.S., Kim D.E., Min D.H. (2010). A Graphene-Based Platform for the Assay of Duplex-DNA Unwinding by Helicase. Angew. Chem. Int. Ed..

[10] Wang Y., Li Z.H., Hu D.H., Lin C.T., Li J.H., Lin Y.H. (2010). Aptamer/Graphene Oxide Nanocomplex for in Situ Molecular Probing in Living Cells. J. Am. Chem. Soc..

[11] He S.J., Song B., Li D., Zhu C.F., Qi W.P., Wen Y.Q., Wang L.H., Song S.P., Fang H.P., Fan C.H. (2010). A graphene nanoprobe for rapid, sensitive, and multicolor fluorescent DNA analysis. Adv. Funct. Mater..

[12] Song Y.J., Qu K.G., Zhao C., Ren J.S., Qu X.G. (2010). Graphene Oxide: Intrinsic Peroxidase Catalytic Activity and Its Application to Glucose Detection. Adv. Mater..

[13] Huang R.C., Chiu W.J., Li Y.J., Huang C.C. (2014). Detection of microRNA in Tumor Cells using Exonuclease III and Graphene Oxide-Regulated Signal Amplification. ACS Appl. Mater. Interfaces.

[14] Huang Y., Yang H.Y., Ai Y. (2015). DNA single-base mismatch study using graphene oxide nanosheets-based fluorometric biosensors. Anal. Chem..

[15] Li J., Huang Y., Wang D., Song B., Li Z., Song S., Wang L., Jiang B., Zhao X., Yan J. (2013). A power-free microfluidic chip for SNP genotyping using graphene oxide and a DNA intercalating dye. Chem. Commun..

[16] Wang H., Chen T., Wu S., Chu X., Yu R. (2012). A novel biosensing strategy for screening G-quadruplex ligands based on graphene oxide sheets. Biosens. Bioelectron..

[17] Wen Y.Q., Xing F.F., He S.J., Song S.P., Wang L.H., Long Y.T., Li D., Fan C.H. (2010). A graphene-based fluorescent nanoprobe for silver(I) ions detection by using graphene oxide and a silver-specific oligonucleotide. Chem. Commun..

[18] Chang H.X., Tang L.H., Wang Y., Jang J.H., Li J.H. (2010). Graphene Fluorescence Resonance Energy Transfer Aptasensor for the Thrombin Detection. Anal. Chem..

[19] Park J.W., Tatavarty R., Kim D.W., Jung H.T., Gu M.B. (2012). Immobilization-free screening of aptamers assisted by graphene oxide. Chem. Commun..

[20] Zhang L.M., Xia J.G., Zhao Q.H., Liu L.W., Zhang Z.J. (2010). Functional graphene oxide as a nanocarrier for controlled loading and targeted delivery of mixed anticancer drugs. Small.

[21] Peng C., Hu W.B., Zhou Y.T., Fan C.H. (2010). Intracellular Imaging with a Graphene-Based Fluorescent Probe. Small.

[22] Qu K., Fang M., Zhang S., Liu H., Zeng X. (2018). A redox conjugated polymer-based all-solid-state reference electrode. Polymers.

[23] Lee D., Sang J., Yoo P., Shin T., Oh K., Park J. (2019). Machine-Washable Smart Textiles with Photothermal and Antibacterial Activities from Nanocomposite Fibers of Conjugated Polymer Nanoparticles and Polyacrylonitrile. Polymers.

[24] Liu F., Zhang Y., Wang H., Zhang S. (2018). Novel Conjugated Polymers Prepared by Direct (Hetero) arylation: An Eco-Friendly Tool for Organic Electronics. Molecules.

[25] Wu J., Yu F., Zhang Z., Chen Y., Du J. (2016). Highly sensitive self-complementary DNA nanoswitches triggered by polyelectrolytes. Nanoscale.

[26] Zhang Z., Wu Y., Yu F., Niu C., Du Z., Chen Y., Du J. (2017). Rapid and annealing-free self-assembly of DNA building blocks for 3D hydrogel chaperoned by cationic comb-type copolymers. J. Biomater. Sci. Polym. Ed..

[27] Zhang Z., Han J.L., Pei Y.X., Fan R.Z., Du J. (2018). Chaperone copolymer assisted aptamer-patterned DNA hydrogels for triggering spatiotemporal release of protein. ACS Appl. Bio Mater..

[28] Maruyama A., Katoh M., Ishihara T., Akaike T. (1997). Comb-type polycations effectively stabilize DNA triplex. Bioconjug. Chem..

